# Postgwas: Advanced GWAS Interpretation in R

**DOI:** 10.1371/journal.pone.0071775

**Published:** 2013-08-19

**Authors:** Milan Hiersche, Frank Rühle, Monika Stoll

**Affiliations:** Leibniz-Institute for Arteriosclerosis Research at the University Muenster, Muenster, Germany; University of California, Riverside, United States of America

## Abstract

We present a comprehensive toolkit for post-processing, visualization and advanced analysis of GWAS results. In the spirit of comparable tools for gene-expression analysis, we attempt to unify and simplify several procedures that are essential for the interpretation of GWAS results. This includes the generation of advanced Manhattan and regional association plots including rare variant display as well as novel interaction network analysis tools for the investigation of systems-biology aspects. Our package supports virtually all model organisms and represents the first cohesive implementation of such tools for the popular language R. Previous software of that range is dispersed over a wide range of platforms and mostly not adaptable for custom work pipelines. We demonstrate the utility of this package by providing an example workflow on a publicly available dataset.

## Introduction

With the implementation of high-density microarray technologies for SNP detection, genome-wide association studies (GWAS) have become a standard method for the identification of susceptibility loci underlying common complex diseases. Despite the wealth of SNPs associated with complex traits, they collectively explain only a small proportion of the phenotypic variance attributable to genetic factors [1]. The remaining missing heritability may be explained by various factors including allelic heterogeneity, independent association of common SNPs or cumulative effects of rare variants in single loci [2], [3] not previously captured on microarrays. In addition, many complex traits exhibit a high degree of locus heterogeneity, with numerous susceptibility loci of moderate effect being scattered over the genome [4]–[7]. This locus heterogeneity may result in related (sub)phenotypes which may or may not share a genomic architecture e.g. as observed for the 5q31 genomic region in chronic inflammatory disorders such as inflammatory bowel disease, atopic dermatitis, rheumatoid arthritis etc [8]–[11]. Therefore, the investigation of related subphenotypes in the context of GWAS is of particular interest in the quest to understand the common genetic architecture underlying complex diseases [12].

In recent years, computational and laboratory techniques have been developed to tackle these obstacles. First, next generation sequencing (NGS) enables the detection of rare variants contributing to the association signals observed in GWAS. Second, the analysis of GWAS results in the context of interaction networks [13], [14] facilitates the prioritization of weaker association signals within biological systems. Such approaches mostly rely on the network guilt by association (GBA) principle [15] and have been implemented recently by DAPPLE [16] and dmGWAS [17]. Related, conventional gene set and pathway enrichment based approaches are summarized in [18]. Additional recently developed methodology encompasses subphenotype comparison and (comparative) rare variant analysis for complex diseases [19]–[21] or systems biology analysis [22]. However, thus far, none of the above mentioned features have been implemented in a single bioinformatics pipeline. The postgwas software package presented here contributes innovative features that support such an analysis of complex traits. In particular, subphenotype comparison and visualization of rare variant data in regional association plots and a flexible interaction network analysis toolset for systems biology analysis have been integrated into the package. At the same time we further simplify, improve and extend the default data processing and visualization methods for GWAS.

Basal statistical analysis of GWAS datasets is well established by software suites like Plink [23] or GenABEL [24], but usually further post-processing steps are required to carry out advanced data analysis, which requires development of additional custom methodology. The presented package aims to avoid repeated implementation of standard data processing procedures by providing appropriate component functions. Commonly performed subsequent steps in GWAS analysis comprise annotation of genes to SNPs, generation of Manhattan plots, regional association plots, derivation of gene-based p-values, GO term enrichment and interaction network analysis. Specific software for application of these tasks exists but is usually scattered over a wide range of web platforms representing individual tools. Hence, data often needs to be reorganized to include all standard features in one comprehensive analysis. Furthermore, the availability of certain data sources is not always guaranteed. Another obstacle posed by web-based tools is a lack of customizability, so that specific adaptations matching the needs of a custom analysis are sometimes hard to achieve. Finally, a number of tools only support a restricted set of model organisms. Since GWAS are more frequently applied to non-human organisms and traits [25], and reference genotype data with recombination and linkage disequilibrium information is available [26], the necessity for universal applicability increases.

The postgwas package aims at a simplified yet customizable workflow that overcomes the obstacles mentioned above. With a single function call, default actions like SNP to gene mapping by LD, construction of regional- and Manhattan plots and basic interaction network research are executed in a pipeline allowing an accelerated interpretation of GWAS results. The major strengths of the package are the applicability for a wide range of organisms, automatic handling of base position and ID translation, usage of linkage disequilibrium data that is directly computed from the study cohort and parallelization features for time-intensive computations. Further, most data sources can be customized or replaced for offline usage.

Besides the unique features, our software adds substantial improvement to the universe of GWAS-affiliated tools by being customizable and open-source, thus giving scientists the best control and transparency on their analysis workflow, especially those working preferentially in R.

## Results

The package is structured into several component functions, each responsible for a certain type of analysis. For the swift use, a superordinate function named *postgwas()* exists, which runs all component functions sequentially without further effort, producing a set of standard plots and interaction network analyses. The input can be GenABEL objects or Plink, GEMMA or FAsT-LMM formatted GWAS result files.

For a sophisticated analysis, each function can be used independently and customized with a wide range of parameters. The full capabilities of the software can be best explored by executing the examples coming with the individual functions, e.g. by stating *example(snp2gene)* in an R session. In addition, a comprehensive tutorial is delivered with the package as a self-executing vignette. In the following paragraphs, we describe the features of each individual tool and its potential for an extended interpretation of GWAS datasets.

### Snp2gene

*Snp2gene()* is a simple to use function that takes a (potentially large) number of SNPs and finds associated genes by proximity or linkage disequilibrium. Although SNP chip vendors often provide gene annotation for their products, these are often incomplete or insufficient with regard to biological reality. Interaction network [14] or pathway analysis [27]–[29] rely heavily on automatic annotation of candidate genes to SNPs and benefit from an accurate annotation. Sometimes it is difficult to assign a single gene to a certain SNP, because several genes may reside in proximity to the SNP in either direction and it is not granted that an observed association signal just refers to the closest gene. Inclusion of multiple genes related to a SNP, i.e. allowing more false positive and reducing false negative annotations, bears the potential to improve findings in computational systems-biology analysis, because these algorithms are usually designed to prioritize from a large number of false positively included genes [30]. Thus, it is reasonable to assume that addition of a small fraction of falsely annotated genes has a less severe impact on the outcome than missing a true causative gene. Annotation by LD as offered by *snp2gene()* allows annotation of multiple genes to a single SNP, however such multi-annotations need to be treated with care because proximate genes tend to share a function and thus cluster together in gene set based analyses, contributing the same association signal multiple times to functional clusters. Nevertheless, it has been shown that for Crohn’s disease, proximate as well as distant effects account for associated SNPs, thus extended annotation can be reasonable [31]. With freedom to adopt multi-annotations, *snp2gene()* allows conventional annotation by proximity as well as reporting the amount of LD between SNPs of interest and its surrounding genes, enabling prospective users to annotate genes based on different measures. Although comparable annotation methods are already available [32], this is to our knowledge the first implementation in R, allowing the use in automated pipelines in this environment.

Besides the assignment of SNPs to genes, mapping of p-values to genes based on SNP associations is another complex task, because the number of statistical tests performed per gene varies with the number of annotated SNPs. We re-implemented two methods for the calculation of gene-wise p-values proposed by Dale Nyholt [33] and Miao-Xin Li et.al. [34] in a function named *gene2p()*. These methods correct for the number of independent tests per gene under consideration of the linkage disequilibrium pattern between annotated SNPs, deriving an effective number of tests per gene together with an appropriately corrected gene-wise p-value.

For all functions, parallelization for multicore architectures has been added to accelerate LD based calculations, however this feature is restricted to UNIX-based machines. Further, our implementation supports a large range of model organisms, can use custom genotype files and, with some effort, even custom background gene sets.

### Gwas2network

*Gwas2network()* is a flexible systems-biology analysis tool that visualizes and decomposes biological interaction data in relation to GWAS results. It aims at detecting ‘cumulative significance’ for GWAS-associated loci i.e. loci that do not reach significance individually but contribute to a common functional mechanism relating to the phenotype at study. When a network module of interactions, which represents such a functional mechanism, contains several moderately associated loci, this can be interpreted as increased evidence for causality.

**Figure 1:**
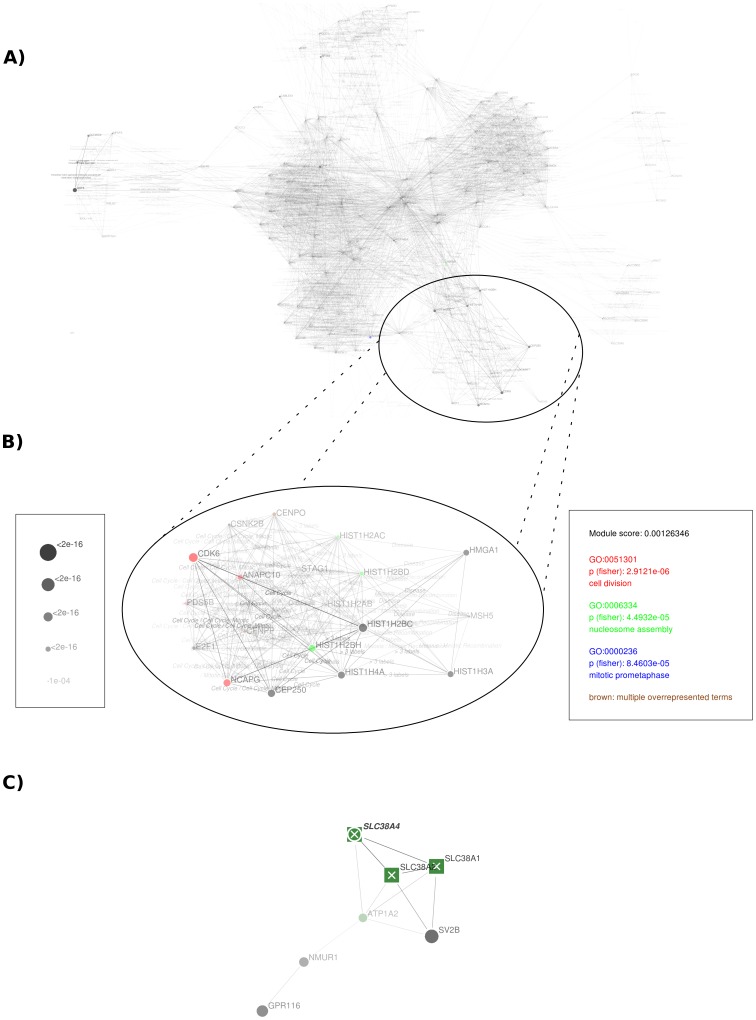
Interaction network analysis in postgwas. Part (A) shows the complete network of genes derived from the human height GWAS dataset [38] using a p-value cutoff at 1×10^−6^, generated by application of the *postgwas()* summary function to the dataset without further customization parameters. Appearance of the network can be modified by using a custom (drag and drop) vertex layout or deactivated edge labels. The edges of this network are formed based on common REACTOME pathway membership, (optionally) labeled by the type of interaction (here: shared pathway name) and weighted by the combined association strength of participating genes. Vertex sizes (and optionally transparency) correspond to the GWAS association p-value. Under consideration of these weights, application of a minimum cut-edge graph partitioning algorithm leads to a decomposition of the global graph into functional subunits with preferential accumulation of well-associated genes within modules. Part (B) shows the first extracted module, exhibiting the strongest evidence for accumulation of low GWAS p-values. This accumulation is reflected by a module score listed in the legend (right box, a lower score corresponds to higher evidence). The major biological functions are identified for each module by GO-term over-representation analysis. The top three over-represented terms are listed in a colorized legend together with the module score. Vertices within the module are colorized according to their membership in over-represented GO terms. Part (C) demonstrates a network analysis for multiple datasets. The module shown has been extracted from a network of GO term similarity between genes from two distinct synthetically generated GWAS datasets. Each dataset corresponds to a vertex shape (squares and circles). For genes occurring in both datasets, vertex shapes are plotted on top of each other in order of their p-value (e.g. SLC38A4). Their label is printed boldface and italic. When a single SNP is annotated to multiple genes (e.g. residing in a larger LD block), these genes are labeled with a cross as shown for three solute carrier genes residing in the same block and having similar molecular functions. Such modules need careful interpretation.

*Gwas2network()* applies a graph partitioning method [35] to a network of biologically related genes as shown in Figure 1– A. The kind of network can be either defined as custom two-column argument or selected from public data on protein-protein interaction, REACTOME pathway membership, domain similarity or GO term similarity networks [36], which are automatically downloaded via the biomart interface. The weight of edges between gene vertices is set to their combined association strength from the GWAS. By default, this is the product of the vertices -log(p), but can be extended with additional measures like biological interaction strength or vertex degree to penalize ‘hub’ vertices. The graph partitioning algorithm then decomposes the entire network into modules by concentrating high-weight edges within modules and minimizing the total weights of between-module edges during the clustering process. This leads to the conclusion that (i) genes with biological relatedness and (ii) reasonable association strengths are combined in each module.

An automated functional classification of the resulting modules can be obtained by enabling an option for GO term overrepresentation analysis within each module. The overrepresentation analysis is performed using Fisher’s exact test as implemented in the *topGO* package [37]. This automatically extracts a universe of all annotated genes (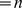) from the gene ontology database, the set of genes related to a GO term (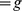), genes within a module (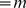) and the overlap between genes associated to the GO term and a module (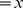). The hypergeometric distribution 
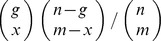
 is then used to assess whether x represents an occurrence of that GO term in the module that is higher than expected by chance. This is done for all GO terms. In addition, GO term decorrelation from the same package is used to consider only the best fitted term within each GO term branch, removing redundancy between reported terms [37]. The resulting p-values are not corrected for multiple testing following the explanations from the *topGO* package. The top three overrepresented GO terms (after decorrelation) are displayed in the legend of each network (see figure 1) and gene vertices within the module are colorized according to the membership within these GO terms.

Secondly, the excess of evidence for association with the phenotype (GWAS p-values) within modules is assessed by a module score. This score is based on the Wilcoxon (Mann-Whitney U) rank sum test, which tests for the hypothesis 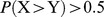. Here, the observed p-values in the entire network are interpreted as a random variable 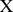, and 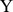 corresponds to p-value observations from the module. By applying the U test, a decision can be made on whether p-values in the module are generally smaller than seen in the entire network and whether this difference is due to chance.

The defined module score, which is set to be equal to the p-value of the U test, serves as a means to rank the obtained modules among each other for their evidence on phenotypic association. By this, this network analysis approach aims at unveiling interactions and biological relatedness between loci at considerable but sub-significant GWAS association levels to allow a reasonable prioritization for further research.

An example application of the presented method is shown in figure 1 building on the human height GWAS meta-analysis dataset [38]. The module with the most reasonable evidence for an accumulation of low GWAS p-values is displayed in the subfigure 1 - B and comprises mainly genes connected by the cell cycle pathway. Correspondingly, overrepresented GO terms comprise cell division and mitotic processes. The investigation of such a module allows the observation of genome wide significant loci in a context with additional, close-to significant genes that could be of additional interest with regard to the ‘guilt by association’ principle (e.g. in figure 1– B the non-significant gene PDS5B (rs17516171) at p = 6.13e-7 might be of additional interest). Furthermore, genes that are not found in the cell cycle pathway but being highly connected to its members are also part of the module (e.g. HIST1H3A). These belong to related (meiotic recombination) or not directly related (disease) pathways and would not have been observed in this context by an analysis focusing on discrete pathways only. Research focusing on pleiotropic genes or yet undiscovered promiscuous functions of genes could potentially benefit from such observations.

Beyond this example based on pathway data, it is, for example, possible to search for modules encapsulating gene families by running the described approach on a gene network linked by shared protein domains. Furthermore, it is possible to merge different kinds of network data, e.g. to investigate protein family membership and protein interaction simultaneously. In comparison to classical gene set enrichment methods, a network-based analysis is able to extend the scope of single biological units and include genes of e.g. related pathways or pathways with overlapping functions, thus providing an extended tool for set-based analyses that, however, requires more consideration during interpretation by the investigator.

As mentioned before, it might be desirable to allow multi-annotations that relate a single SNP to several genes. Because this introduces a potential bias, vertices that receive their p-value from a multi-annotated SNP are tagged by a cross in our visualization. Another indicator for emergence of the module by a single SNP association (or at least fewer associations than total genes in the module) are identical vertex sizes between genes in a module as exemplarily shown in figure 1– C, where three green vertices are tagged and have the same vertex size (synthetic data). Such modules have to be treated with care. The *gwas2network()* function produces a subsidiary multi-annotation file for the lookup and confirmation of such cases. Recommended usage of gene-based p-values improves the accuracy of p-value assignments, however does not fully circumvent the problem that multiple functionally related genes may be associated through LD. Thus a single associated locus may contribute several times (via multiple genes from the same block) to a pathway or network module.

The applicability to diverse species and subphenotype comparability as an important general feature of the package is also included in this function. For subphenotype comparison, multiple GWAS datasets can be merged and appear with different vertex shapes in the network as shown in figure 1– C. Ideally, shared and discriminating functional mechanisms between phenotypes can be identified either by spotting boldface genes or identification of clusters comprising different vertex shapes. Since the statistical power of a GWAS impacts the expected strength of association, it is recommended to use the subphenotype comparison only on equally powered studies to ensure comparability of p-values.

### Regionalplot and Manhattanplot

**Figure 2:**
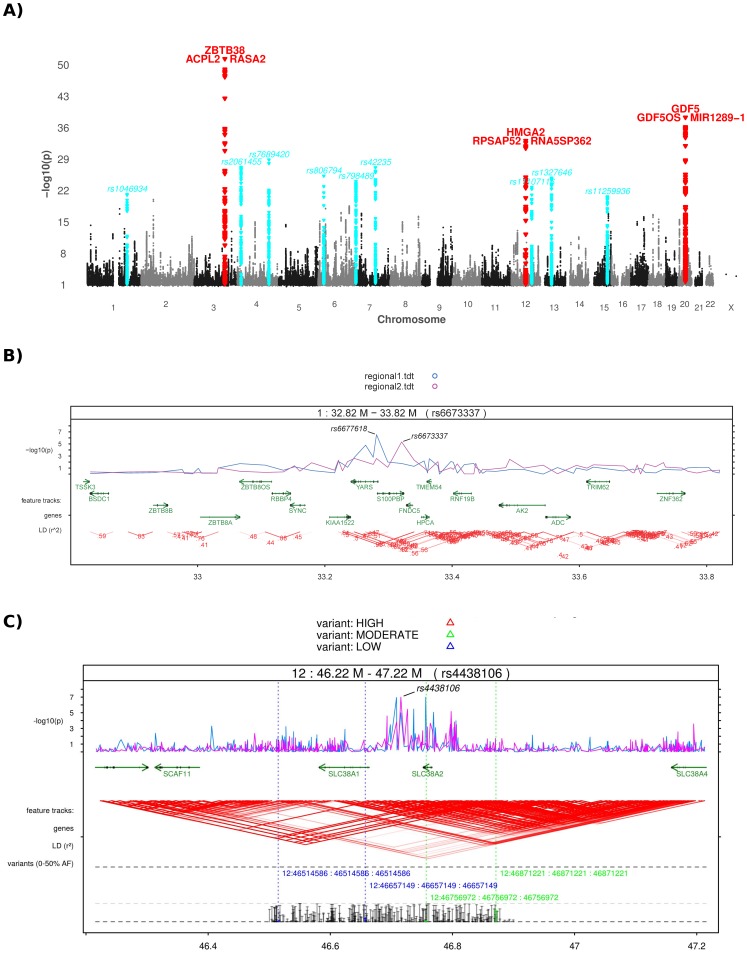
Manhattan and regional plots of random datasets. Part (A) shows a conventional manhattan plot as produced with the default options on an artificial GWAS dataset. Peak SNPs that exceed genomewide significance are colored in red and are annotated with the closest genes (covering genes are placed above for intragenic SNPs, up- and downstream gene left and right). A second threshold is set by default for suggestive association at P<1*10^−5^ with gene annotations in blue. Annotation text can be deactivated or replaced with the identifiers of peak SNPs. Part (B) and (C) display regional plots with different unique capabilities. Both contain tracks showing the association p-value graph, genes with strand and exon information and a triangle LD plot where the color intensity reflects the r^2^ correlation between SNPs. Identifiers of queried SNPs are automatically annotated to the pvalue graph but can take custom annotation text as well. The LD plot uses either custom genotype files or HapMap data and is available for arbitrary large regions. In (B), r^2^ values have additionally been annotated to the LD triangles and we compare p-value graphs of two distinct datasets (color code listed in the legend). In (C), rare variant information from a resequencing study is included in a track at the bottom, showing allele frequencies in a histogram at the very bottom and identifier, position (original and remapped) and calibration lines for selected variants above. Only de novo variants are displayed here using filter settings on the histogram. A second filter has been set on the position information display to include only variants of certain predicted functional effects (determined by SnpEff). The color code for the variant effect is listed in the plot legend above.

The *regionalplot()* and *manhattanplot()* functions comprise common state-of the art characteristics as comparable tools, as shown in exemplary plots in figure 2. Nevertheless, we also provide several novel features.

First, it is possible to plot a large number of loci in a single searchable pdf file. Having the genomic context of the top associated loci always at hand, literature research and interpretation of GWAS results were substantially simplified in our own studies [39]. In later stages, when the original GWAS dataset has been retired, the existence of a comprehensive summary file encompassing all loci of suggestive or moderate significance can be very useful, e.g. to check for the presence of a novel published candidate gene in the own dataset.

Second, we included the ability to plot a data track with resequencing results from a variant call format (.vcf) file. This track consists of a histogram with allele frequencies and optionally position and predicted effect information for selected variants. Variant selection is currently based on regular expression filtering in the INFO and ID columns of the supplied vcf file for the sake of generality. This allows to selectively display e.g. only de novo variants or those with specific predicted functional effects listed in the INFO column, e.g. as generated by *SnpEff* [40]. Another filter can be set on the allele frequency field, limiting the display to variants of a certain rarity (figure 2– C). In addition, we have implemented an experimental feature to display comparative histograms between two vcf files, e.g. cases and controls, but currently this works for datasets with identical minor alleles only. To our knowledge this rare variant track is the first visualization utility that allows the comparison of common versus rare variant association. The comparison of signals derived from common and rare SNPs is interesting under the assumption that multiple causative rare variants are in cumulative LD with a common marker SNP or reside on the same haplotype, respectively (allelic heterogeneity). Several statistical algorithms that analyze such accumulation of rare variants have been proposed [41] and the visualization of such regions is now feasible with the presented *regionalplot()* function.

Third, by using a line graph for association p-values, it is possible to include multiple datasets in a single regional plot, each displayed in a different color (figure 2– B). This features the inter-phenotype comparison of association signals from distinct GWAS and enables the comparative display of discovery and replication sets of multi-staged GWAS. In a broader sense, it is also possible to discover pleiotropic genes by searching for overlapping association peaks for distinct phenotypes. This feature may be simultaneously analyzed within networks as outlined in figure 1– C.

Finally, the investigation of long range LD block structures is possible by varying the region window sizes. For very large regions at megabase scale, there is an option to restrict the LD calculation to a user defined number of SNPs that are selected evenly distributed over the region. The original block structure is very well preserved in our experimental plots at moderate interleaving settings (data not shown).

With our implementation of regional plots in R, we try to offer the well-established strengths of R plotting functions to the user, e.g. by providing scaling parameters. Further improvements are customizable data sources, e.g. using custom genotype files for LD calculation or HapMap genotype retrieval, access to internal data via buffer variables, etc. We aim to increase the flexibility of our regional plots in comparison to web-based tools by using a tracks concept. Beside activation or deactivation of default tracks, it is possible to reserve empty space for drawing custom data which offers potential for custom extensions.

### Implementation Details

**Figure 3:**
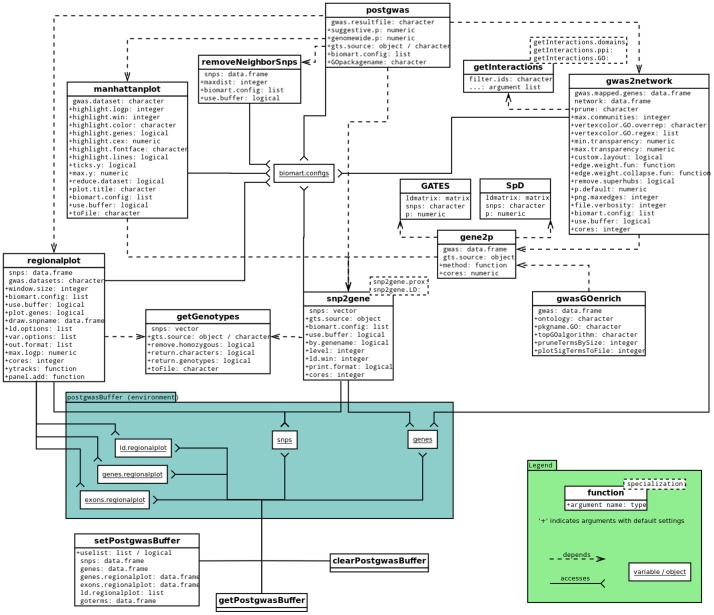
Diagram of functions, parameters and dependencies in the postgwas package. Individual functions are represented by white boxes divided into an upper part listing the function name and a lower part containing argument names and types. Arguments preceded by a ‘+’ sign are optional and contain default values. Dashed lines denote a ‘used by’ relation: For example, the superordinate function *postgwas* calls *removeNeighborSnps*, *gwas2network*, *snp2gene*, *manhattanplot* and *regionalplot*. Only functions that are exported from the package (documented and visible to the user) are shown. Non-segmented boxes denote variables from a special environment that are used by internal functions (indicated by solid connectors) and available to the user through publicly visible getter/setter functions.

From a design perspective, the major advantage of our package in comparison to similar (web-based) software is the availability of source code and the possibility to concatenate customizable functions in a custom analysis pipeline. The degree of customizability is further enhanced by a flexible control over data sources using custom biomart configurations and the possibility to view, supply and manipulate annotation buffer data. The overall package structure is listed in figure 3, and implementation details for critical items are discussed in the following paragraphs.

### Genomic Positions and Data Sources

A major challenge in the design of annotation tools is the correct mapping of genomic positions. For the whole package, we use as reference genome and base positions the current contents of biomart databases (default is ENSEMBL but can principally be any other biomart). Thus, all data e.g. from GWAS output is automatically mapped and corresponds to the current biomart release. By selecing preconfigured or supplying custom biomart configuration lists, various model organisms and different kinds of IDs can be annotated (e.g. entrez or ensembl gene IDs for genes, protein accession etc).

Technically, all SNPs are mapped to biomart positions by their reference SNP ID (dbSNP), and genes are retrieved from the same data source. SNPs that cannot be mapped to a new position (e.g. rare or de novo variants) get an imputed position assigned by using the offset of the nearest mapped SNP.

### Custom Data and Buffer Variables

Throughout the package, there is the possibility to store downloaded or calculated data in local buffer variables. Re-running the function will use the buffer data if existent, which saves time and bandwidth for repeated calls with slightly changed parameters (e.g. tuning graphical appearances). Further, all source data can be viewed by the user if needed, and even modified or extended when it is incomplete or wrong (which can sometimes happen with newly discovered genes, inclusion of specific splice variants, inconsistent positions etc). The probably largest benefit of using buffer data is that it can completely replace the automated usage of web data sources if needed. This is of special interest for bioinformatics applications in general due to the high variability of data sources used and turnover of data contained, which often decreases the lifetime of developed algorithms and software. By storing the R session together with the buffer variables to file, it is always possible to reproduce the obtained results of an analysis even when the web data sources changed within time. Also, when source data for specific cases is not available by automatic retrieval, it can be obtained manually and supplied as buffer variable without use of the web access options.

However, using buffer data is intended for experienced users because of the obvious danger to accidentally use outdated buffer data from a previous run that was generated under different conditions or in a different analysis. Therefore this feature is deactivated by default.

### SNP to Gene Annotation

For annotation by proximity, we use a sequentialization technique on chromosomes that allows the annotation of all queried SNPs at once in a vectorized fashion. For sequentialized chromosomes, the *findInterval()* function from the *base* package rapidly returns the index of the closest gene for all query SNPs at once. We also consider overlapping genes in the annotation process which exist more frequently than expected by us in advance.

Annotation by LD uses either custom genotype files in ped/map format, GenABEL genotype objects or automatic retrieval from HapMap for human data. For each SNP, gene positions are retrieved within 1 MB distance to determine LD with the query SNP. When custom genotypes are used as source data, gene positions are shifted by the offset between positions given in the source data and biomart positions. Using these gene boundaries, all intragenic SNPs are determined and downscaled to at most 100 SNPs per gene (evenly distributed). Genotypes for this selection are then retrieved and used in pairwise LD calculation with the query SNP.

### Network Processing

To allow a simple use of the package, we have pre-implemented functions for retrieval of pathway, protein-protein interaction, protein domain similarity and gene ontology term similarity data [36], but principally, arbitrary interactions can be passed as a data frame argument to the function. Basically, such network data is defined as two-column argument containing either gene IDs or symbols for interacting genes. Optional columns like edge weight or labels can be included. Secondly, a list of GWAS-derived genes has to be defined which can be obtained by running the *snp2gene()* and optionally *gene2p()* functions. Then, loop-edges in the network are removed and duplicate edges combined, where duplicates with identical labels are eliminated and different labels collapsed to a single label. The label collapsing feature enables the use of different kinds of networks in a single analysis. Afterwards, the network is truncated to gene vertices from the GWAS-derived list or, optionally, also preserving ‘shared interactors’ (genes that are not listed in the GWAS dataset but connected by two such genes). Lastly, ubiquituously interacting genes (‘superhubs’) can be removed to increase the specificity. In our experiments, we found that superhub vertices tend to form modules in the network regardless of linking only genes with low association strength. Thus, we established the possibility to remove such hubs, given they are not annotated in the GWAS list. A second mechanism to control for over-proportionally interacting genes is to correct for vertex degree in the edge weight function.

One of the major strength of functional programming in R is the use of functions as values. In order to allow custom definition of edge weights, user defined function can be passed as arguments that calculate weights based on vertex p, degree and fixed weight from the network data.

This has a direct influence on the clustering results and allows different ways of interpretation. For example, increasing the influence of vertex degree on edge weight will emphasize the aggregation of heavily interconnected genes in favor to strongly coupled vertices by GWAS association.

### Parallelization

Runtime-intensive operations have been parallelized for Linux architectures. We use the package named *parallel* that is based on forking the R process for parallel computation. Therefore, memory consumption increases with the same rate as computation speed. Currently, parallelization is offered for data extraction from large genotype files and for LD calculations. This can be useful for the generation of a larger set of regional plots with a window size exceeding 1 MB or calculation of gene-wise p-values, but for common tasks parallelization will not be necessary.

### Regionalplot Tracks Concept

One of the design objectives during the development of the *regionalplot()* function was flexibility and extensibility. Beside a rich parametrization in general, we again provide a function argument that takes a user defined function as value which is called during the plotting process. In the spirit of custom panel functions in the *lattice* package, this lets the user implicitly draw into the panel area. Within the body of the self-defined panel function, the user has access to all relevant internal data so that it is possible to add content to the panel in dependence of existing data.

Although it is possible to draw anywhere using the custom panel function (e.g. p-value graph area), there is a data frame that defines y axis boundaries for each track. By adding a row with additional y boundary specification, it is possible to reserve blank space in the panel for adding a separate track with custom data. All relevant internal variables are passed to the panel function as arguments and are thus accessible.

An example application would be to draw rectangles into the gene track at operon positions for bacterial organisms. The user then has to supply a data frame with correctly mapped operon bounds and apply the *panel.rect()* function from *lattice* package, using the y positions from the tracks object.

### Regionalplot Rare Variant Display

Data extraction for the rare variant track uses the *RSamtools* [42] package. This includes the possibility to download genotypes of selected variants from the 1000 Genomes project for comparison with the own dataset which might be implemented in a future version. Unknown positions of rare variants are estimated as explained in the ‘genomic positions’ paragraph. The histogram display and frequency pruning functionalities always refer to the minor allele which is determined after reading the vcf file.

### Modular Architecture and Utility Functions

Shared functionality between the main parts of the package has been decomposed into standalone functions that might be of interest for the user. There are functions to retrieve genotypes, genes and SNPs and remap them to current base positions or different IDs, calculate LD between a larger set of SNPs in parallel, and select representative SNPs within a window from a set of larger SNPs. The latter is for example useful to identify the lead SNP of a region of association.

### Examples

All major functions are provided with examples demonstrating their features. They are called from within R by issuing *example(functionName)* after loading the package with *library(postgwas)*, The placeholder ‘functionName’ has to be replaced by one of the postgwas functions. A complete list of exported functions is obtained by stating *objects(“package:postgwas”)*. Also, a tutorial can be accessed by stating *vignette(“postgwas”)*.

### Availability and Future Directions

The package has been deposited on CRAN for download or direct installation using the *install.packages()* function.

There are several features that are desirable for future development. For example, the snp2gene assignment could be improved by inclusion of expression boundary information. Furthermore, the graph partitioning approach that assigns each vertex to exactly one module could occasionally be replaced with a soft clustering method allowing multi-assignments of vertices to modules. As pleiotropy is a frequent mechanism in biology, genes will often be found to be connected to several different modules, each representing a functional mechanism that the gene belongs to. Such constellations could be more efficiently captured by soft clustering approaches. Finally, there are many potentially useful additions imaginable for the regional association plots, for example an additional optional track displaying methylation site status.
